# Contributions to expenditure in endoscopic stone management: a costly process

**DOI:** 10.1007/s00240-022-01344-z

**Published:** 2022-07-08

**Authors:** Romy Mondschein, Damien Bolton, Sarah Tan, Minh Hang Vu, Philip McCahy

**Affiliations:** 1 Department of Surgery (Urology), Monash Health, Berwick, VIC Australia; 2 Austin Health, Urology, Heidelberg, Melbourne, VIC Australia; 3 Department of Medicine, Monash University, Clayton, VIC Australia; 4 School of Clinical Sciences, Monash University, Clayton, VIC Australia

**Keywords:** Ureteropyeloscopy, Cost, Urolithiasis, Laser-lithotripsy

## Abstract

No comprehensive cost estimates exist for performing ureteropyeloscopy (URS), which is increasingly utilised as a treatment of upper tract urolithiasis in Australia. To estimate expenditure associated with URS in an Australian public hospital setting and determine factors contributing to increased cost. Patients who underwent flexible URS for urolithiasis over a 2-year period at a Victorian public health site were included. Data describing demographics, stone factors, disposable equipment and admission length were retrospectively collected. Procedures were performed using reusable flexible scopes. Previously validated costing models for cystoscopic stent extraction, theatre and recovery per hour and ward admission were used to attach cost to individual episodes. The cost of emergency stent insertion was beyond the scope of this study. 222 patients underwent URS; the combined total number of procedures was 539, comprising 202 stent extractions and 115 stent insertions in addition to 222 URS. Mean procedural cost was $2885 (range $1380–$4900). Mean episode cost excluding emergency stent insertion was $3510 (range $1555–$7140). A combination of flexible scopes, operative time and disposable equipment accounted for nearly 90% of the total procedural cost. Significant cost is associated with URS for treatment of renal and ureteric stones. A large burden of the cost is time in theatre, equipment and the need for multiple associated procedures per episode. Utilising other available treatments such as extracorporeal shockwave therapy (SWL) where appropriate may reduce the financial burden of URS and associated procedures.

## Introduction

URS has been available for treating intrarenal and ureteric stones in Australia for over 30 years. Procedure numbers have been growing rapidly [1], while other standard treatments (extracorporeal shockwave lithotripsy (SWL) and percutaneous nephrolithotomy (PCNL)) have remained static or declined [2]. The trend towards minimally invasive treatment has been driven by advancing scope technology, lasers and adjunct equipment [3]. However, significant variability exists in the equipment utilised at different hospitals across Australia. Considerations for upgrading or changing equipment include accessibility, safety, efficacy and cost. With the incidence of urolithiasis increasing globally [4], attributing cost to what is now the most common procedure conducted for stone disease in Australia is highly relevant.

There are many variables when estimating cost of URS. Flexible scopes may be disposable or reusable acquisition and sterilisation costs must both be considered [5]. Recently, lasers producing up to 120 watts have become available. These ‘high-power’ lasers may improve efficiency of stone destruction, result in fragments and dust that can be passed spontaneously and reduce use of basket devices [6]. However, the advantages of high-power lasers in a clinical setting remain uncertain, and their effect on operative time is unknown; high-level comparative evidence translating laboratory observations to the clinical environment is lacking [7]. Use of disposable equipment including baskets and access sheaths is variable and depends on stone location, composition, surgeon preference and patient factors. In addition to the cost of URS, episode costs vary depending on other associated procedures. Primary URS may not be possible; stent placement electively or in the emergency setting is often required [8]. Post-procedural stent placement necessitates cystoscopic extraction, although ‘stent on a string’ is utilised by some health services. Large stones may require multiple URS to clear stone burden. Although laser URS is recognised for its efficacy in achieving adequate stone clearance [2], up to date Australian data on whether this is being achieved after a single URS procedure is lacking. Finally, theatre time and hospital expenses are significant contributors to the overall cost of any procedure [9]. Time efficiency is a target of operating theatres across Australia as the financial implications of poorly utilised theatre time are well documented [9].

The aim of this study was to determine the mean procedural cost of URS conducted for treatment renal calculi in an Australian public hospital setting. Further to the procedural cost of URS, secondary aims were to estimate the total hospital episode cost associated with URS procedures, and to determine significant contributions to cost along the treatment pathway.

## Materials and methods

Consecutive patients who underwent URS over a 2-year period at one site of a major public Victorian health service, after being assessed as inappropriate for medical expulsive therapy, were included. Patient demographics, stone burden and location were recorded. Stone burden was calculated from maximal stone diameter measured on CT scan. Where multiple stones were present, the sum of each stone’s diameter was recorded. Time in theatre, time in recovery and use of disposable equipment were recorded. Previously validated costing models for theatre and recovery per hour were used to attach cost to individual procedures [10]. This was added to (i) the cost of specific disposable equipment used in each procedure including laser, basket, access sheath and stent and (ii) the cost of capital equipment including 120 W laser, image intensifier (II) and flexible ureteropyeloscope. These components formed the total procedure cost. The 120 W laser and II purchase prices ($230,000 and $170,000, respectively) were divided by their expected life span of 10 years and 10% added for annual maintenance to obtain the annual cost. For the laser, this figure was divided by all laser cases performed annually including rigid ureteroscopy, HOLEP and percutaneous nephrolithotomy to obtain a per-case cost. The II case-cost was calculated with inclusion of total annual cases including non-urological surgeries. Flexible ureteroscopes were purchased for $20,000 per scope and have an expected lifespan of 20 uses.

Cost was attributed to admission length and cystoscopic stent extraction based on previous modelling [10]. These components were added to the total procedural cost, to obtain a total episode cost for each patient. Stent insertion prior to URS was recorded; however, these costs were predicted to be highly variable and beyond the scope of the study. Therefore, the cost of stent insertion was omitted from total episode cost. Data on additional URS procedures were collected to assess single-URS stone clearance rates. Costs are in 2018 A$. Descriptive and summary statistics were obtained for demographic and cost parameters; multivariable linear and logistic regression and student’s t-test was performed to assess contributions to procedural and over-all episode cost. Stata 16.0 (StataCorp. 2019. Stata Statistical Software: Release 16. College Station, TX: StataCorp LLC) was used for this analysis.

## Results

### Demographics and stone characteristics (Table 1)

**Table 1:** Cohort demographics, stone characteristics, procedures and admission details

Demographics	Mean (range, standard deviation), *n* = 222
Age (years)	53.0 (18.0–83.0, 15.0)
BMI (kg/m^2^)	30.0 (18.8–58.4, 6.2)
Stone burden (mm)	10.6 (2.0–65.0, 7.6)

	*n*, % (222)
Stone location	
Ureteric	19 (8.6)
Pelviureteric junction (PUJ)	25 (11.3)
Intrarenal	110 (49.5)
Multiple locations	62 (27.9)
Data not available	6 (2.7)
Procedures per episode (*n*)	
1 Primary URS	4 (1.8)
2 Stent insertion and URS	16 (7.2)
3 Stent insertion and URS and stent extraction	99 (44.6)
2 URS and stent extraction	103 (46.4)
Total procedures	539

Procedures included in episode costs^a^	*n*
URS	222
Cystoscopic stent extraction	202
Total procedures costed	424

Admission Length (nights)	*n*, %
0	78 (35.3)
1	128 (54.3)
2	17 (7.7)
3–4	5 (2.3)
> 4	1 (0.5)

^a^Cost of episode-associated emergency or elective stent insertion (*n* = 115) was not calculated

**Fig. 1:**
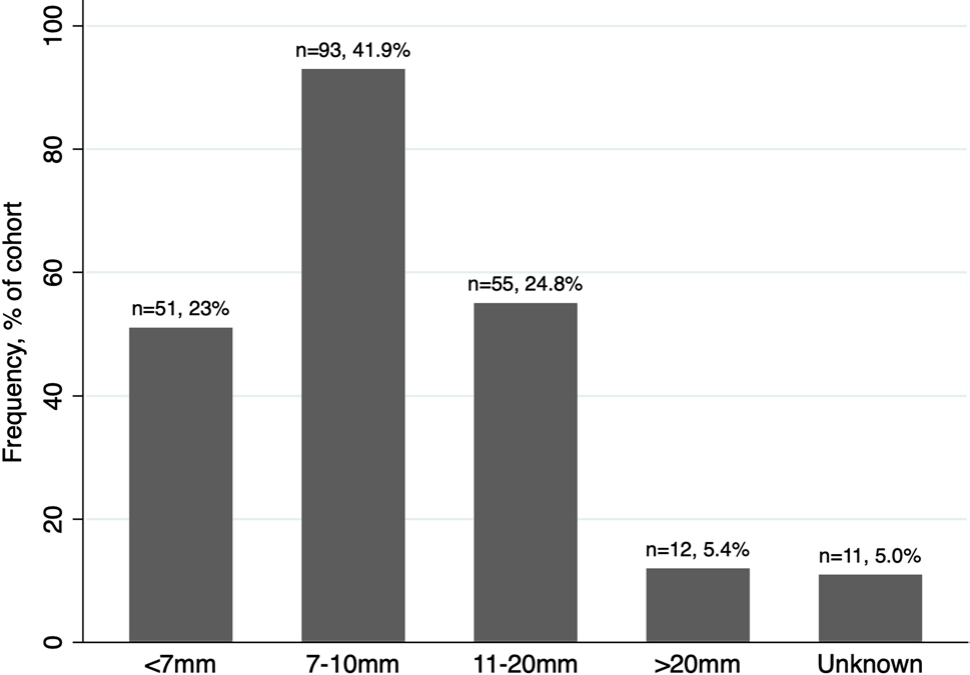
Stone size categories identified within the URS cohort

Median patient age of the 222 participants was 53.5 years, and median BMI was 29 kg/m^2^. Median stone diameter was 9 mm; 67% (*n* = 148) of calculi fell into the 7–20 mm size category (Fig. 1.) Most frequently, stones were located intra-renally (49.5%, *n* = 110); however, 62 patients (27.9%) had stones in multiple locations (intrarenal and ureteric or PUJ).

### URS procedural cost

This cost was calculated for each procedure using the figures in Table 2. Total procedural cost comprised the costs for theatre time, recovery time, disposable items, laser, image intensifier, radiographer and flexible pyeloscope described above. The mean total procedural cost was $2885.0 (SD $545, range $1380–$4900).

**Table 2:** URS management components and their corresponding cost, used in procedural and episode cost calculations

Item/unit	Cost
**Procedure cost components**	
Theatre per hour	$898.43
Recovery per hour	$299.9
Basket	$320
Laser fibre	$310
Access sheath	$150
Stent (inserted at URS)	$175
120 W laser per case	$74.0
Image intensifier per case	$13.7
Radiographer per hour	$39.74
Flexible Pyeloscope	$1000
**Episode cost components**	
Overnight admission	$327.2
Cystoscopic stent extraction	$374.41

### URS episode cost

This cost incorporated the procedural cost described above, in addition to ward admission costs for each case based on their length of stay and associated cystoscopic stent extraction listed in Table 2. The mean total episode cost was $3510 (SD $680, range $1555–$7140).

35% (*n* = 78) of patients were managed as day cases and 54.3% (*n* = 120) were admitted overnight. A small proportion of patients stayed two nights (7.7%, *n* = 17) or longer (*n* = 6, 2.7%). Cystoscopic stent extraction was applicable to 91.0% (*n* = 202) of the cohort (Table 1).

### Contributions to cost

A 1-min increase in theatre time was associated with a cost increase of $16 (*p* < 0.001, CI 14.7–17.9); this was very close to the predicted association of cost and theatre time per minute based on the model used (Table 2). Stone size was associated with a cost of $15.0 per 1 mm increase in calculus diameter (*p* = 0.001, CI $6.0–$25.0), although this relationship became insignificant after adjusting for operative time, which was significantly affected by stone size (Table 3). Disposable items contributed disproportionately to procedural cost compared to the known cost of the item; this relationship was also attenuated following adjustment for time and co-used items (e.g. access sheaths were associated with stent insertion, OR 3.80, *p* = 0.007, CI 1.40–10.1) (Table 4). Stone location did not demonstrate significant relationships with either procedural cost or operative time.

**Table 3:** Stone size categories and their corresponding effect on operative time

Stone size	Minutes in theatre (mean)	*p* value	CI
< 7 mm	53	< 0.001	46–60
7–10 mm	60	0.131	51–69
11–20 mm	67	0.005	57–77
> 20 mm	77	0.004	61–93

**Table 4:** Cohort utilisation of disposable equipment, presented with the unadjusted cost of the item, item cost following modelling and each item’s association with operative time

Item	Uptake (*n* = 222)	Item cost	Cost association (*p* value, CI)	Operative time association (minutes) (*p* value, CI)
Basket	98 (44%)	$320	$356 *p* < 0.001, $301–411	7 *p* = 0.068, − 0.5 to 14
Laser Fibre	190 (86%)	$310	$357 *p* < 0.001, $276–$438	11 *p* = 0.029, 1–21
Access Sheath	184 (83%)	$150	$197 *p* < 0.001, $123–$272	− 7 *p* = 0.885, − 10 to 9
Stent^a^	202 (91%)	$175	$80 *p* = 0.13, $23–$184	12 *p* = 0.067, − 1 to 24

^a^After adjusting for basket, laser, access sheath, stent insertion and minutes in theatre

Overall, the three main contributors to procedural cost were scopes, theatre time and disposable items, accounting for a combined total of 89.6% of the total mean procedural cost. Scopes ($1000) as a fixed cost accounted for 34.6% of mean procedural cost. Mean theatre cost was $897 (median $823, interquartile range $614–$1123), which represented 31.0% of the mean total procedural cost. Mean cost of disposable items was $692 (median $635, range $0–$955), which represented 24.0% of the calculated mean procedural cost.

Stent insertion, resulting cystoscopic stent removal, was associated with an episode cost $753 higher compared to no stent insertion (*p* < 0.001, $455–$1052). Day cases were associated with a $400 reduction in total episode costs compared to those who stayed 1–3 nights (Table 1).

### Post-operative outcomes and further procedures

Fifteen complications (6.8%) were identified ranging from post-operative sepsis to ureteric mucosal abrasions. Complications were associated with a mean episode cost of $3960 (SD $487, CI $3690–$4230), which, on average, was $482 more than episodes that did not involve complications (*p* = 0.008, CI $128–$836). 186 patients (83.8%) underwent one URS and associated procedures resulting in cleared stone burden. 36 patients (14.4%) went on to have further URS. Six patients subsequently underwent PCNL (Table 5).

**Table 5:** Further procedures undergone by the URS cohort including further endoscopic treatment and percutaneous nephrolithotomy

Patients (*n*, %)	Number of URS	PCNL
186 (83.8)	1	4
32 (14.4)	2	2
3 (1.4)	3	-
1 (0.5)	4	–
Total		
222	263	6

## Discussion

By using our previously validated hospital cost model, we have estimated that treating a renal stone with URS costs on average A$2885 per procedure and $3510 per episode, with wide variation depending on patient complications and length of stay. The fixed cost of flexible, re-usable ureteropyeloscopes accounted for around a third of mean procedural cost. Time in theatre attracted nearly $16 per minute mostly due to staff wages [10] and as a result accounted for a third of the mean procedural cost. Stone size also contributed significantly to overall cost as larger stones were associated with increased operative time. Laser fibres, stent insertion, baskets and access sheaths were individually associated with procedural costs that were elevated disproportionately to the known cost of each item. These inflated costs were attenuated after adjusting for operative time, with which these items were significantly associated, and equipment co-use. Disposable items represented around a quarter of the mean procedural cost.

Re-usable flexible scopes were utilised at our centre. With an associated purchase cost of around A$20,000 and an expected lifespan of 20 cases, these scopes contributed significantly to the procedural cost of URS. Estimates vary regarding case numbers before requiring repair, ranging from 5 to 28 in the literature [11]. Tosoian et al. conducted a cost-analysis of URS cases which included the cost of scope repair. They reported a higher per-case cost of URS compared to our findings. Much of the difference was attributable to scope repair costs, which were found to be US$605 per case due to a scope-life of around 10 cases [11]. Although we did not calculate the precise scope life, our estimate is conservative and demonstrates that extending scope life may significantly decrease costs attributed to URS. While some studies have identified that single-use scopes exceed the cost of reusable equipment, others have found no difference. In addition, the cost of single use scopes is likely to decrease over time. Viability is likely to depend on case-volume and scope life at individual centres [12].

Based on stone size, location and patient factors, over 50% of our patient cohort could have been appropriate for primary treatment with SWL, which is an available alternative in both the elective and emergency setting at our centre. There is increasing reinterest in SWL as primary treatment for sub-1 cm stones [13]. A recent UK-based study found that URS was more costly than ESWL for treating stones < 1 cm when considering outcomes including stone free rates, re-treatment rates, complications and adverse events. Their analysis identified URS as the more costly treatment even if initial SWL treatments had a 40% probability of success [13]. Geraghty et al. found URS to be less costly in their metanalysis of 12 studies comparing SWL and URS. However, reporting on cost inclusions for each study protocol revealed that most centres conducted SWL in the theatre setting under general anaesthetic (minimum 7/12 studies) [14]. At our centre, SWL is conducted without general anaesthesia in a dedicated space within Diagnostic Imaging making it a much cheaper option. Clearly, costs of stone treatments vary between centres depending on protocols, equipment and treatment success. At our centre, stone clearance with a single URS treatment was high (84%). However, based on our URS cost data, the SWL protocol used at our centre and cost comparison from similar centres utilising non-operative treatments could significantly reduce the cost of treating stones in selected patients at our centre. Alternatives to operative management in a COVID era is a pertinent goal; theatre resources and staff are a finite resource during these times.

Laser type is also an important consideration when considering cost-efficiency of URS procedures. Despite promising laboratory results, there is currently no high-level evidence that high-power lasers improve stone destruction time that results shorter operative time and therefore lower procedural cost [7]. In addition, a recent study investigating operative time and cost-efficacy of the Moses holmium laser found that despite its potential advantages in reducing lasing time, this did not translate to lower procedural cost as a result of technology and equipment expenditure [15]. In fact, use of the 35 W Holmium laser was found to be the cheaper alternative in this study. There may be a role for new laser technology in reducing the costs associated with URS procedures in the future. However, neither Moses technology nor high-power lasers have strong evidence associating reduced operative costs with their use; our estimate of URS procedural cost likely represents minimum expenditure.

A potential confounder of this study was the lack of cost information regarding stent insertion procedures. Commonly at our centre stents are placed in the emergency setting, where costs can include after hours and emergency staff, inpatient admission of variable length due to emergency theatre demands and intensive care unit admissions. Stent insertion episode cost is highly variable with components outside the scope of this study. A separate study is planned to address this topic. Despite this, emergency stent cost estimation will only serve to increase the already elevated cost of URS episodes. SWL is expected to be a much less costly treatment at our centre.

## Conclusion

Significant cost is associated with URS for treatment of renal and ureteric stones. A large burden of the cost is time in theatre and the need for multiple associated procedures in a manner that is much more common with routine ureteroscopic management of upper tract calculi. Utilising other available treatments such as SWL, PCNL or medical dissolution therapy where appropriate may reduce the financial burden of urinary stone disease, as well as reducing staff and resource burden associated with operative theatres in a COVID-era.
